# Viruses of the Fall Armyworm *Spodoptera frugiperda*: A Review with Prospects for Biological Control

**DOI:** 10.3390/v13112220

**Published:** 2021-11-04

**Authors:** Ahmed G. Hussain, Jörg T. Wennmann, Georg Goergen, Astrid Bryon, Vera I.D. Ros

**Affiliations:** 1 Laboratory of Virology, Wageningen University and Research, Droevendaalsesteeg 1, 6708 PB Wageningen, The Netherlands; ahmedgbenga.hussain@wur.nl (A.G.H.); astrid.bryon@wur.nl (A.B.); 2 Julius Kühn Institute (JKI)—Federal Research Centre for Cultivated Plants, Institute for Biological Control, Heinrichstr. 243, 64287 Darmstadt, Germany; Joerg.Wennmann@julius-kuehn.de; 3 International Institute of Tropical Agriculture (IITA), Biological Control Centre for Africa, Cotonou 08 BP 0932, Benin; G.Goergen@cgiar.org

**Keywords:** *Spodoptera frugiperda*, FAW, viruses, baculovirus, SfMNPV, biological control

## Abstract

The fall armyworm (FAW), *Spodoptera frugiperda*, is a native pest species in the Western hemisphere. Since it was first reported in Africa in 2016, FAW has spread throughout the African continent and is now also present in several countries in Asia as well as Australia. The invasion of FAW in these areas has led to a high yield reduction in crops, leading to huge economic losses. FAW management options in the newly invaded areas are limited and mainly rely on the use of synthetic pesticides. Since there is a risk of resistance development against pesticides in addition to the negative environmental and human health impacts, other effective, sustainable, and cost-efficient control alternatives are desired. Insect pathogenic viruses fulfil these criteria as they are usually effective and highly host-specific with no significant harmful effect on beneficial insects and non-target organisms. In this review, we discuss all viruses known from FAW and their potential to be used for biological control. We specifically focus on baculoviruses and describe the recent advancements in the use of baculoviruses for biological control in the native geographic origin of FAW, and their potential use in the newly invaded areas. Finally, we identify current knowledge gaps and suggest new avenues for productive research on the use of viruses as a biopesticide against FAW.

## 1. Introduction

The fall armyworm (FAW) *Spodoptera frugiperda* (J. E. Smith) (Lepidoptera: Noctuidae) is an economically important pest species native to the Americas [1]. It is a highly polyphagous pest species, feeding on more than 350 plants species, including important staple crops, such as maize, rice, sorghum, and soybean [2]. *Spodoptera frugiperda* causes 15–100% yield loss (depending on the extent of infestation) in its native range in the Americas [3]. Historically, populations of *S. frugiperda* have been divided into two morphologically identical but genetically distinct groups, known as the corn strain, with feeding preference for corn, sorghum, and other large grasses and the rice strain that preferentially feeds on rice, bermudagrass, and other smaller grasses [4,5]. FAW invasion on the African continent was first reported in 2016 on the mainland of West Africa (Nigeria, Benin, and Togo) and on the islands of São Tomé and Príncipe [6], and has since spread across most countries in sub-Saharan Africa [7]. The highly migratory pest has also invaded the Asia-Pacific region, with reports of its new occurrence in India, China, Bangladesh, Myanmar, Malaysia, Indonesia, Thailand, the Philippines, and Sri Lanka, amongst others [8,9,10,11], and in Australia and New Zealand [7]. More recently, FAW has been reported on the Canary Islands of Spain [12], where it is currently restricted to maize crops. This represents the first report of the invasive pest in a European territory. At present, FAW has spread to over 70 countries from its native Western hemisphere [7]. In Africa, FAW has the potential to reduce maize yields by 8.3 to 20.6 million metric tons per year, accounting for 21–53% of annual maize production. The annual economic loss in maize as a result of the FAW outbreak is estimated to be worth between US $2.48 billion and US $6.19 billion, based on just 12 of Africa’s maize-producing countries [8,13]. A more recent study has estimated such economic loss to reach US $9.4 billion for Africa [14].

The exact migration patterns of FAW into the newly invaded areas in the Eastern hemisphere is currently under debate. Earlier studies based on the mitochondrial cytochrome oxidase subunit I (*COI*) gene and the sex-linked triosephosphate isomerase (*Tpi*) gene demonstrated that FAW populations in Africa share similarities with the populations in Florida and the Greater Antilles, suggesting that the likely site of introduction into Africa is from these areas [15,16]. This finding was further strengthened in other studies that showed that the FAW populations in Africa, India, and Southeast Asia share a common site of introduction [17,18]. However, a recent study using a whole genome sequencing approach found a panmictic *S. frugiperda* population structure, and suggested multiple sites of introduction into the Eastern hemisphere [19]. Nonetheless, the FAW poses a great economic threat to crop production and livelihood, especially among smallholder farmers in newly invaded areas. Many rural farmers in China and parts of Africa are facing a reduced profitability in the production of maize due to the purchase of chemical pesticides to control FAW [20,21].

Current management options of FAW mainly involve the use of chemical pesticides, which have been practiced for several decades. However, the risk of resistance development and the undesirable health and environmental effects pose a major concern. Field-evolved resistance has been reported against main insecticide groups in Mexico, Puerto-Rico, and the United States [22,23], and more recently against emamectin benzoate [24], which is one of the most frequently used pesticides against FAW in Africa. FAW has developed resistance to at least 30 pesticide active ingredients in North and South America [25]. This has redirected the focus towards other effective, more sustainable, and environmentally friendly alternatives. In its native Western hemisphere, FAW is routinely controlled using transgenic crops expressing *Bacillus thuringiensis* (Bt) toxins that are lethal to FAW (genetically modified, GMO crops). However, FAW has developed resistance against more than one commercial GMO crop variety and causes significant damages to these crops [26,27]. Other alternatives are currently being explored, including the use of biological control agents, such as entomopathogenic fungi, viruses, nematodes, bacteria (including Bt spray formulations), plant extracts, and parasitoids, and methods, such as the use of pheromone traps and push-pull technologies [8,28,29,30]. A promising alternative is the use of viruses for the control of FAW. Some insect viruses are virulent, restricted to a narrow host range, and have no environmental impacts compared to chemical pesticides [31,32,33]. Recent technical innovations have increased the availability of virus products on the market for the control of a variety of insect pests worldwide. Similarly, Spodoptera frugiperda multiple nucleopolyhedrovirus (SfMNPV), a baculovirus infecting *S. frugiperda*, has become commercially available and is to date registered in some countries for the control of *S. frugiperda* [34,35]. Apart from baculoviruses, some other virus species are known from lepidopteran insects, mainly discovered in caterpillar mass rearing or in cell culture (lepidopteran cell lines). In this review, we give an overview of all viruses described from FAW and their potential use for biological control. In addition, we discuss recent advances in the use of baculoviruses for biological control in the native area of origin of FAW, as well as the potential application of those baculoviruses in newly invaded areas. Lastly, we identify existing knowledge gaps and propose new directions for future research on the use of viruses as biocontrol agents against FAW.

## 2. Current Status of Viruses in the Control of FAW

Most of our current knowledge on insect viruses results from studying viruses that threaten mass rearing of economically important insects, including the silkworm *Bombyx mori*, the two-spotted cricket *Gryllus bimaculatus*, and the house cricket *Acheta domesticus* [36], or viruses that have potential as biocontrol agents of important agricultural pests, such as the cotton bollworm *Helicoverpa zea* and defoliators of forest trees including the gypsy moth *Lymantria dispar* [37]. Those viruses include ascoviruses, baculoviruses, and densoviruses [33,38,39,40]. Since the past decade, novel viruses of lepidopteran species are being discovered in large-scale sequencing studies (detailed description below). In this section, we mention all virus families found associated with *S. frugiperda*, describe their key biological characteristics and symptoms on the susceptible insect stages, and mention their potential as biological control agents.

### 2.1. Ascoviruses

Ascoviruses (family *Ascoviridae*) are large, enveloped, double-stranded DNA (dsDNA) viruses with a genome size ranging from 100–200 kilo base pairs (kbp) [41]. Infection by ascoviruses is mainly restricted to species of the Noctuidae family in the order Lepidoptera [33,39] and only infect the larvae of these species. Parasitoids are the natural agent of transmission of ascoviruses in the field. Ascovirus infection triggers the formation of virion-containing vesicles in the hemolymph of infected larvae and gives the hemolymph a milky appearance, a distinctive symptom of the disease (Table 1) [39,42]. The circulation of virions and vesicles in the hemolymph facilitates mechanical transmission of the virus from a diseased to a healthy larvae or pupae by endoparasitic wasps during oviposition [39]. Ascoviruses cause chronic to fatal disease and infected larvae show stunted growth, difficulty in molting, and eventual death. Ascovirus infections are found in all larval stages in natural populations of *S. frugiperda* [43,44]. Hamm et al. [43] reported the first incidence of ascovirus infection in natural FAW populations found in the USA. Additionally, two ascovirus-infected *S. frugiperda* larvae were retrieved in field surveys in Mexico characterized by the presence of vesicles [45]. Despite their high virulence, there have been no records of their use in biological pest control. Transmission by parasitoids presents a major limitation in the use of ascoviruses for biological control programs since *per os* infection of larvae is rarely effective [33,43]. Ascoviruses are unable to establish *per os* infection due to the inability to completely overcome the barrier of the insect midgut [46]. The combination of ascoviruses with other insect pathogens that can lyse the insect midgut and cause the initial infection could allow the establishment of an ascovirus infection. Mixtures of Heliothis virescens ascovirus isolates (HvAV-3h and HvAV-3j) with *B*. *thuringiensis kurstaki* (Btk) was effective to lyse the midgut of the lepidopteran species *Mythimna separata,* and *S. litura*, leading to increased mortality compared to the control group (Btk only), with the exception of *Helicoverpa armigera* and *S. frugiperda*, where low mortality was observed [47]. The low mortality observed for *S. frugiperda* larvae infected with the mixture could be a consequence of the failure of Btk to lyse the midgut cells (*S. frugiperda* has been previously shown to exhibit low susceptibility to the Btk strain Cry1AC [48,49]), or of the inability of the Heliothis virescens ascoviruses to infect *S. frugiperda*. Nevertheless, the combination strategy could potentially improve the *per os* infection challenge of ascoviruses, thereby providing a promising control alternative.

### 2.2. Baculoviruses

Baculoviruses (family *Baculoviridae*) are large, circular dsDNA viruses with genome size ranging from 80–180 kbp and infect the larval stages of mainly lepidopteran species, many of agricultural importance [55,56,57]. The family is divided into four genera: *Alphabaculovirus* (nucleopolyhedroviruses (NPVs) infecting lepidopteran species), *Betabaculovirus* (granuloviruses (GVs) infecting lepidopteran species)*, Gammabaculovirus* (NPVs infecting hymenopteran species), and *Deltabaculovirus* (NPV infecting a dipteran species) [58]. Baculoviruses have a bi-phasic infection cycle with two types of enveloped and rod-shaped nucleocapsids (virions): occlusion-derived viruses (ODVs, initial infection in the midgut) and budded viruses (BVs, spread within the insect). The ODVs are embedded in occlusion bodies (OBs; also known as polyhedra) [55,57,58]. The OBs surround, protect, and confer environmental stability to the infectious virions against biotic and abiotic factors, such as UV light [56,58,59]. As a result, OBs allow for relatively long-term storage and for spraying of baculoviruses in aqueous suspensions [35,60]. Two morphological types of baculoviruses are distinguished: NPVs and GVs, that differ in the viral protein making up the crystalline matrix of the OBs, polyhedrin for NPVs and granulin for GVs [57,58]. Baculoviruses have been extensively studied for their application in biological control (as biopesticides) and for their use in biotechnological applications as expression vectors for in vitro protein production and as a delivery vector in gene therapy studies in mammals [34,55].

Baculoviruses and their hosts share a close co-evolution, which is reflected by a mostly very narrow host range restricted to single or closely related host species [57,61,62,63]. The high specificity allows a targeted and specific insect pest control with no negative impact on humans, the environment, and beneficial insects in contrast to chemical pesticides [35,64]. A typical example of an extremely narrow host range is found for alphabaculoviruses infecting *Spodoptera* species, such as SfMNPV, Spodoptera exigua MNPV (SeMNPV), Spodoptera littoralis nucleopolyhedrovirus (SpliNPV), and Spodoptera litura NPV (SpltNPV; Table 2). In addition, the suitability of baculoviruses in integrated pest management (IPM) programs together with other control agents have rendered them as a highly attractive insect pest control alternative to chemical pesticides. Ultimately, humans exploited these properties to use baculoviruses against economically important pests. Most baculoviruses cause lethal infections to insects and infect the larval stages of insect species. Infected larvae show lethargic behavior, a swollen body with whitish pale-discoloration, death, and often liquefaction of the larvae (Table 1) [65,66]. Additionally, some baculoviruses induce behavior manipulations in caterpillars, which includes hyperactivity and climbing to the top of the plant or tree before liquefaction [67,68].

SfMNPV is the main viral candidate used worldwide in the biological control of *S. frugiperda* [72,73,74,75]. Several SfMNPV isolates are used, some of which cause high larval mortality in *S. frugiperda* [66,76,77,78,79]. In diseased populations, dead caterpillars are an important source of inoculum for the occurrence and maintenance of epizootics [34,35]. Epizootics are desired for biological control since dead caterpillars can facilitate the spread of a virus to healthy non-infected ones. Other baculoviruses are known to infect *S. frugiperda*. SpliNPV is a baculovirus used to control the African cotton leafworm, *S. littoralis*, and was effective in tests against *S. frugiperda* with up to 60% larval mortality [80]. SpliNPV is currently marketed for the biological control of FAW [80,81]. Although other baculovirus isolates can provide an alternative to control *S. frugiperda*, often inter-host effectivity is lower and therefore obtaining local baculovirus isolates of SfMNPV and/or Spodoptera frugiperda granulovirus (SfGV) is crucial to effectively control local FAW populations.

### 2.3. Rhabdoviruses

Rhabdoviruses (family *Rhabdoviridae*) are negative-sense ssRNA viruses with a variable genome size [82,83]. They possess a wide host range, infecting vertebrates, invertebrates, and plants [82]. Many rhabdoviruses infecting vertebrates and plants are vectored by arthropods [82]. Few rhabdoviruses have been isolated from insects and from insect cell lines, mainly from the order Diptera [82,83]. Three insect-specific rhabdoviruses (sigma viruses) are currently described from *Drosophila melanogaster*, *Drosophila affinis*, and *Drosophila obscura*, respectively [83]. Using next-generation sequencing (NGS) and bioinformatic analyses, a rhabdovirus (Sf-rhabdovirus) was identified in the *S. frugiperda* Sf9 cell line, representing the first reported rhabdovirus in a lepidopteran cell line (Table 1) [53]. Sf-rhabdovirus is more closely related to plant rhabdoviruses than to vertebrate or invertebrate rhabdoviruses. Sf-rhabdovirus was found permanently infecting Sf9 cells and the virus sequence was also detected in a parental Sf cell line, Sf21 [53]. In addition, Sf-rhabdovirus is infectious to the High Five cell line from the noctuid *Trichoplusia ni* and the SL2 cell line from *D. melanogaster* but is not infectious to human cell lines [53,84]. Subsequently, two lepidopteran-associated new sigma-like viruses were discovered from RNA-seq data [85]. More recently, the presence of genetically diverse isolates of Sf-rhabdoviruses was identified in naturally occurring adult *S. frugiperda* populations [84]. Nonetheless, no study has so far demonstrated the presence of the rhabdoviruses in any of the larval stages of FAW.

### 2.4. Other Virus Families

Other viruses that infect lepidopteran insect species, and may be found to infect *S. frugiperda*, include DNA viruses, such as iridoviruses, entomopoxviruses, densoviruses, and nudiviruses, and RNA viruses, such as iflaviruses, cypoviruses, tetraviruses, dicistroviruses, and nodaviruses [31,33,86]. Some of these viruses have been successfully applied in biological control programs against pests of important crops and plants. Notable examples include the use of Oryctes rhinoceros nudivirus (OrNV) to control the coconut beetle, *O. rhinoceros*, in coconut and oil-palm plantations [31,87]. In addition, a commercial cypovirus product (Matsukemin^®^) has also been produced and registered in Japan against pine moth, *Dendrolimus spectabilis* [37]. Furthermore, some densoviruses have been successfully used to control insect pests [88]. Junonia coenia densovirus (JcDV), originally isolated from the buckeye butterfly *J. coenia*, can orally infect *S. frugiperda* larvae by rapidly binding to the peritrophic matrix of the insect midgut through interaction with different glycans, including chitin and glycoproteins [89]. In addition, JcDV also interferes with midgut gene expression, which leads to dysfunction of the gut barrier [89]. JcDV rescued from lysate of insect cells transfected with JcDV infectious clone caused mortality in second instar of *S. frugiperda* [51]. In addition, *S. frugiperda* larvae were susceptible to oral infection with JcDV propagated on *S. litura* larvae, although it required a higher lethal dose (1.76 × 10^8^ viral genome copies per larva), compared to *S. litura* (7.39 × 10^7^ viral genome copies per larva) and *H. armigera* (9.71 × 10^7^ viral genome copies per larva) [51]. The study demonstrates the potential of JcDV as a biological control candidate to control *S. frugiperda* [51]. However, the family *Parvoviridae* also contains other important vertebrate viruses, such as vertebrate porcine parvovirus, that share sequence homologies with densoviruses, thereby raising safety concerns for the use of densoviruses as biopesticides [88]. To our knowledge, there has been no report of naturally occurring *S. frugiperda* infection by these densoviruses.

### 2.5. NGS to Discover New Viruses

Our current knowledge of insect viruses originates from research into viruses that pose a threat to insect mass rearing or viruses with biocontrol potential. However, over the past decade, there has been a tremendous increase in insect virus discovery, due to NGS approaches and metagenomics [54,83,85,90,91,92,93,94,95,96]. The use of NGS is becoming the most popular modern scientific tool to uncover the viruses present in the genome, transcriptome, and small RNA sequences of several invertebrates and arthropods, especially insects [54,83,85,91,92,97]. Several existing and novel viruses have been (re)discovered and described from different arthropods owing to high-throughput NGS and sophisticated bioinformatic analysis. These tools have presented a plethora of opportunities to uncover the “hidden treasures” in the genomic and transcriptomic sequence data of these organisms. The current largest single study discovered over 1000 RNA viruses present in the transcriptome of more than 220 invertebrate species [95]. Among insects, the virome of mosquitoes has been well studied, with new viruses described [98,99].

In species of the order Lepidoptera, two iflaviruses, Spodoptera exigua iflavirus 1 (SeIV-1) and 2 (SeIV-2), have been discovered from the transcriptomic data of *S. exigua* [92,97]. The SeIV-1 sequences were not present in an *S. frugiperda* laboratory colony that had been reared next to *S. exigua* colonies for more than 20 generations [92]. The iflaviruses that were isolated from *S. exigua* appear to be present in a covert (non-symptomatic) state in different *S. exigua* laboratory colonies and field-collected individuals [92,100,101]. The role of these iflaviruses is still not fully understood and is currently being studied. However, recent studies demonstrated that the coinfection of *S. exigua* with baculovirus SeMNPV and iflaviruses (SeIV-1 and SeIV-2) influences host growth and reproduction and reduces the median lethal concentration of SeMNPV OBs compared to larvae infected with SeMNPV alone [100,102].

Similarly, three novel partiti-like viruses (Table 1; SEIV1, SEIV2, and SEIV3) were discovered in the lepidopteran species *Spodoptera exempta* from NGS data and bioinformatic analysis of all life stages of the pest (eggs, larvae pupae, and adults) [54]. The three partiti-like viruses were found coinfecting field populations of *S. exempta* and their coding sequences were determined [54]. Based on the amino acid sequences of the peptides, SEIV2 and SEIV3 were found to be similar; therefore, SEIV1 and SEIV2 were used for infection and host range studies. Both SEIV1 and SEIV2 were successfully transmitted via microinjection in four lepidopteran species, including *S. exempta*, *S. frugiperda*, *S. littoralis*, and *H. armigera* [54]. Furthermore, SEIV1 and SEIV2 were transmitted vertically from infected females to their progeny in the four lepidopteran species and infected individuals had delayed growth and reduced fertility compared to the uninfected control [54]. In their natural host, *S. exempta*, the partiti-like viruses act as mutualistic symbionts, by decreasing the susceptibility of *S. exempta* to a baculovirus Spodoptera exempta nucleopolyhedrovirus (SpexNPV), but appear parasitic in the non-natural host, *S. frugiperda,* making the larvae more susceptible to a baculovirus SfMNPV challenge [54].

Additional studies are needed to increase our knowledge on the role of newly discovered viruses in their hosts. In addition, uncovering the virome of important lepidopteran species, like *S. frugiperda*, will not only show the complex network of viruses present but also help us understand the functional relationship of these virus(es) to host survival, distribution, and the interaction between the viruses and other microorganisms present in the insects. Furthermore, the uncovered viruses could also play a role in biological control. Either as antagonists or as synergists, reducing or increasing the effectiveness of biocontrol agents like SfMNPV. Additionally, they could also serve as potential biological control agents.

## 3. SfMNPV: The Most Promising Viral Candidate for the Biological Control of FAW

Over the past decades, several SfMNPV isolates have been obtained from dead *S. frugiperda* larvae collected in crop fields and pastures in the Americas (Table 3) [76,103,104,105], with some developed into commercial biopesticides. Since the first report of the invasion of FAW into several countries in the Eastern hemisphere, efforts have been made to collect and characterize effective natural enemies that may have co-invaded with FAW into the new areas. Baculoviruses (SfMNPV and SfGV) are one of those natural enemies that have been found in association with the pest in its native origin [45,79,106,107]. Knowledge on the occurrence of SfMNPV and SfGV in association with the invading population of FAW is still limited. However, naturally occurring field isolates of SfMNPV have been found in newly invaded areas like China [108], India [50,109,110], and Nigeria [111]. Initial characterization of an SfMNPV field isolate from China (SfHub) shows that there are two naturally occurring genotypes (SfHub-A and -E) that differ in their biological characteristics [108].

Commercial isolates of SfMNPV have been registered and successfully employed to control *S. frugiperda* in North and South America, and more recently in some African and Asian countries (Table 4) [28,32,35,72]. To date, only two SfGV isolates have been isolated from *S. frugiperda* [118,120]. SfGVs are characterized by a relatively slow speed of kill, requiring up to 24 days to kill *S. frugiperda* larvae [120]. The virulence (in terms of lethal concentration and speed of kill) of the different isolates of SfMNPV and SfGV vary widely. One of the main reasons for the differences in virulence is the variations in some of the genes that make up the genomes of the different isolates [78,121,122]. Aside from the core genes that most baculoviruses share, variations arising from insertions and deletions and single nucleotide polymorphisms in parts of the genome have been reported among different isolates [76,123,124]. In addition, the virulence of SfMNPV isolates also depends on the *S. frugiperda* strains present in the field. Bioassay experiments showed different levels of susceptibility of the corn- and rice-strain larvae to the same SfMNPV isolates, with the corn strain showing a broader range of susceptibility to the SfMNPV isolates (in terms of lethal concentration and speed of kill) compared to the rice strain [79].

### 3.1. In Vitro and In Vivo Plaque Purification

The analysis of the genotypic composition of field-collected SfMNPV isolates plays an important role in their description and is a key feature in the successful control of FAW. While the detection of SfMNPV intra-isolate variation was mainly performed by restriction endonuclease patterns [106,129,130], whole genome sequencing approaches became the standard for detecting genotypic variation in SfMNPV samples [78,104,111,122,124]. It is suggested that a mixture of genotypes in a single field isolate usually reduces the isolate’s potency and prolongs the time to death, thereby ensuring a higher production of OBs [115]. It is therefore desirable to purify the single genotypes from such field isolates and test the characteristics of each genotype separately. Studies have exploited the use of in vivo (passage in live larvae) or in vitro (in competent insect cell lines) plaque purification techniques to purify the different genotypes and used DNA restriction endonuclease analysis to characterize these [76,104,108,114,115]. One of the genotypes derived from a field isolate from United State isolate 3AP2 possesses desired traits like fast killing properties and is currently commercialized for usage in America and in some countries in Africa and Asia [66,72]. Separation of single genotypes from field isolates with plaque assays can provide an efficient method to find naturally occurring variants of the field isolate with more desirable traits for biological control.

Deletions in parts of the genomes of SfMNPV isolates is a phenomenon commonly found in natural isolates and such deletions may affect the biological properties of the isolates [61,104,113,122]. Similar deletions have been found in SeMNPV isolates and seem to represent an evolutionary mechanism generating and sustaining those deletion mutants optimizing virus survival and/or transmission [131]. Comparison of three geographically distinct isolates of SfMNPV (SfMNPV-NIC-B, 3AP2, and 19) with different levels of virulence showed that deletion in the region around the gene encoding ecdysteroid UDP-glucosyl transferase (*egt*) is the main difference between SfMNPV-3AP2 and NIC-B, while small deletions and point mutations in SfMNPV-19 distinguish this isolate from SfMNPV-NIC-B [122]. Masson et al. [124] also found deletions in the *egt* region as one of the most frequent structural variations among five SfMNPV isolates. Deletion in the *egt* region of the SfMNPV-3AP2 isolate was found to be linked to its fast killing ability [104,121]. EGT can prolong infection and increase the number of progeny OBs produced in each infected insect [132,133]. Therefore, deletions in part of this gene shortens the infection period, thereby killing the larvae faster. In addition, deletions in part of the *egt* region in the field isolate of SfMNPV-NIC and its reconstituted mutants significantly increased the speed of kill compared to the field isolate with the *egt* region intact [121]. In contrast, SfMNPV isolates 459 and 1197 were recently described as fast killing isolates, with infected neonate larvae dying less than 56 h post infection in the tested larvae groups [79]. Analysis of the genomes of isolate 459 and 1197 showed that their *egt* region is intact [79]. This suggests that other factors may be involved in the fast killing properties of SfMNPV isolates. The speed of kill is a highly important viral property in biological control to reduce the feeding damage of caterpillars. It would therefore be interesting to investigate other factors that could influence this property. In general, natural genotypes obtained via plaque purification from field isolates of SfMNPV that possess fast killing properties normally produce less OBs compared to the field isolate where they are purified [66,108,115].

### 3.2. Specificity of SfMNPV

Most known baculoviruses infect one or few related species. Some exceptions include the well-studied viruses Autographa californica MNIPV (AcMNPV) and Mamestra brassicae MNPV (MbMNPV), which are generalists with broad host ranges causing larval mortality in a wide range of insect species belonging to different families [134,135]. In contrast, SfMNPV has a very narrow host range and infection of larvae is mainly restricted to *S. frugiperda* [61]. Additionally, as with other baculoviruses, SfMNPV does not infect non-target organisms, such as pollinators or beneficial organisms. For example, field-scale studies showed that SfMNPV had no adverse effect on non-target and beneficial organisms, such as predatory earwigs and beetles, in contrast to chemical pesticides, such as chlorpyrifos, which had a detrimental effect on the natural enemies [136,137,138]. This implies that when SfMNPV is sprayed in new geographic regions, such as Africa or Asia, no/few impact is expected on the new (unknown) environment. Furthermore, SfMNPV is compatible with and successfully employed in IPM systems in combination with other control strategies, including spinosad [139,140], Bt foliar sprays [141], and transgenic Bt expression [142,143,144]. These properties have endeared SfMNPV as the most suitable candidate for the biological control of *S. frugiperda*. In addition to inter-host specificity, intra-host population specificity of baculovirus isolates is also a common phenomenon in many baculovirus–host interactions [145]. This specificity has been observed for the SfMNPV–*S. frugiperda* complex. Earlier studies have shown that the susceptibility of *S. frugiperda* populations to SfMNPV isolates is dependent on the geographical location from where they both originate [107]. The intra-host specificity has been established in studies in which SfMNPV isolated from a local population induces higher mortality than SfMNPV isolated from a different geographical location. The Colombian SfMNPV isolate (SfCol) was more effective against a *S. frugiperda* population from Colombia, and is 12-fold more pathogenic compared to another isolate from Nicaragua (SfNIC) [76]. Similarly, *S. frugiperda* populations from Honduras were more susceptible to neighboring isolates of SfMNPV from Nicaragua (SfNIC) and the USA (Sf-US), than to another geographically distant isolate from Argentina (Sf-Ar), which required a 14-fold median lethal concentration to achieve the same mortality [106]. In the context of the newly invaded areas, finding SfMNPV isolates associated with the invading host population is crucial for effective control in these regions.

### 3.3. Phylogenetic Analysis of SfMNPV and SfGV Isolates

**Figure 1:**
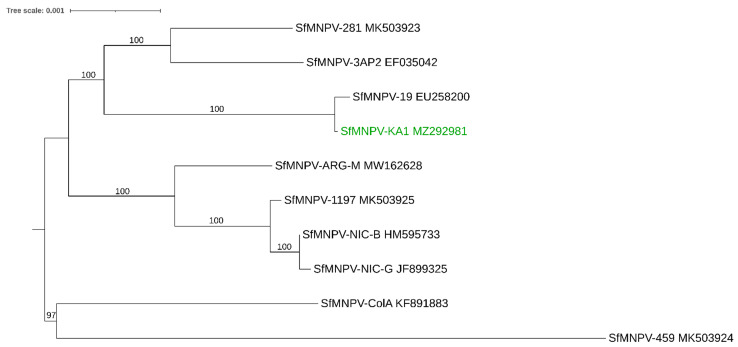
Evolutionary relationship of SfMNPV isolates with completely sequenced genomes. The newly described SfMNPV from Africa (SfMNPV-KA1) [111] is represented in the figure with a green font. The maximum likelihood phylogeny is shown. Numbers indicate maximum likelihood bootstrap values (1000 replicates). The scale bar represents 0.001 expected substitutions per site. Methodology: Whole genome sequences were obtained from GenBank (see accession numbers in the Figure). Both SfMNPV-ColA and SfMNPV-459 contain a region starting near the 3′ end of the *chitinase* gene until the 5′ end of the *gp37* gene that seems to be acquired by recombination from another alphavirus species (closest match to Spodoptera litura NPVII, EU780426; see Popham et al. [79]. This region was excluded from both isolates (position 21642 until 24292 for SfMNPV-ColA; position 21621 until 24276 for SfMNPV-459). Sequences were aligned using MAFFT version 7 with default settings [147]. PAUP* version 4.0a [148] was used to select the optimal evolution model by critically evaluating the selected parameters [149]. Maximum likelihood analysis (heuristic search, 1000 bootstrap replicates) was performed in PAUP, using a submodel of the General Time Reversible Model with invariable sites and a gamma distribution of among-site rate variation (GTR + I + G) with rate class ‘abcdea’.

The complete genomes of 11 baculoviruses isolated from *S. frugiperda* have been published on the NCBI database. Nine of these isolates are nucleopolyhedroviruses (genus *Alphabaculovirus*): isolate 3AP2, accession number EF035042 [104]; isolate 19, accession number EU258200 [78]; isolate NIC-B, accession number HM595733 [122]; isolate ColA, accession number KF891883 [123]; isolate NIC-G accession number JF899325 [146]; isolate ARG-M, accession number MW162628 [124]; isolate 281, accession number MK503923 [79]; isolate 459, accession number MK503924 [79]; isolate 1197 accession number MK503925 [79], while two are granuloviruses (genus *Betabaculovirus*): isolate VG008, accession number KM371112 [119] and isolate ARG, accession number MH170055 [118]. In addition, despite being the continent with the first report of FAW in the Eastern Hemisphere, to our knowledge, only one naturally occurring isolate of SfMNPV has been found in Africa so far (in Nigeria, West Africa). The whole genome of the isolate (SfMNPV-KA1) has been recently sequenced and uploaded on the NCBI database under the accession number MZ292981 [111]. To understand the relationship between the different SfMNPV isolates, phylogenetic inference was conducted using the whole genome of all the SfMNPV isolates available on the NCBI database (Figure 1). Two well-supported clusters are observed, one containing an isolate from Colombia (ColA) and from the United States (459) and the other containing all other isolates (from Argentina, Brazil, Nicaragua, Nigeria, and the United States). The recently sequenced Nigerian SfMNPV-KA1 isolate is highly related to SfMNPV-19 from Brazil (Figure 1). There are several possible ways to explain how the virus has followed its host to Africa (and other parts of the world): (a) it could have followed FAW via infected larvae, (b) as a covert infection in adults or other live stages, or (c) through the use of commercial SfMNPV formulations, as commercial formulations were available in the Americas when FAW invaded Africa. However, the last hypothesis could be ruled out since there were no sprays of commercial SfMNPV formulations applied in the field when the larvae were collected [111].

The two SfGV isolates, SfGV-VG008 and SfGV-Arg, have genome sizes of 140,913 bp and 139,812 bp respectively, with SfGV-Arg being 1101 bp smaller due to the lack of an SfGV-VG008 open reading frame 084 (ORF084) homologue, which encodes for *lef-7* and few indels throughout the genome [118,119]. Although initially 146 ORFs were annotated for SfGV-VG008 [115], later it was found that all but one (ORF084) of the 151 annotated ORFs for SfGV-Arg were also found for VG008 [118]. Both isolates encode four *chitinases* and two *enhancins*, which are important virulent factors associated with insecticidal properties [118,119]. The genomes of the two isolates have been extensively compared elsewhere [118].

## 4. Challenges of Using Baculoviruses for Biological Control

The application of baculoviruses for biological control is facing technical and social challenges. One of the technical challenges is the reduced efficacy of baculoviruses against target pests compared to chemical pesticides. Field-derived virus isolates often require a higher concentration and take more time to kill pests [35,40]. Although there is limited research data available, it is important to note that the FAW strain present in the field may influence the efficacy of the baculovirus isolate. An interesting way to search for more virulent isolates is by purifying single genotypes derived from field isolates (see Section 3.1 on in vitro and in vivo plaque purification). There are at least two reports of single genotypes obtained from field-derived SfMNPV isolates that are more virulent (in terms of lethal concentration and speed of kill) than the field isolate from where they were obtained [66,115]. The increased virulence is attributed to natural deletions in parts of the genome [104,121]. In a recent study, mixtures of NPV and GV isolates, SfCol and SfGV-VG008, have shown promising results to control second instar larvae of FAW, with increased larvae mortality compared to assays with single isolates [65]. The increased virulence of the mixture of the two isolates is attributed to the presence of *enhancin* genes in the SfGV, which are absent in the SfCol isolate, thereby providing a promising bio-insecticide ability. Enhancins function by improving the penetration of ODVs into the peritrophic membrane (PM) by proteolyzing insect intestinal mucin (a major mucinous component of the PM) during infection [55,150]. Nonetheless, timely detection of FAW infestation in the field (early instar stages) and early application of baculovirus isolates could increase the overall treatment efficiency. Another characteristic that limits the efficacy of baculoviruses is the susceptibility of their OBs to ultraviolet (UV) radiation, which damages the viral DNA. This reduces their efficacy in the field and their half-life. As a result, storage conditions to maintain high virulence are crucial and may play a role, particularly in climatic conditions, such as those found in sub-Saharan Africa. Microencapsulation can be used to increase the storage and stability of SfMNPV isolates by protecting the formulated OBs against UV radiation, thereby prolonging their usage and potency in the field [66,151,152,153]. The microencapsulation technique involves the coating of small solid particles (in this case OBs) in a thin layer of coating materials to protect the OBs from adverse environmental conditions. The OBs can then be formulated in the form of an emulsion, spray drying, and wettable powders [66,154,155]. Microencapsulation formulations of OBs into wettable powders uses methacrylic acid polymer, such as Eudragit S100^®^, a pH-dependent polymer that completely solubilizes at high pH (9 and above), which corresponds to the pH of the insect midgut [151,152,154]. Behle and Popham [66] demonstrated that the half-life for the efficacy of encapsulated virus isolate 3AP2 was >26.7 h, compared with a half-life of >7.5 h for the unformulated virus when exposed to natural sunlight under field conditions. Similarly, Castillejos et al. [156] also found an increase in the persistence of the activity of the viral inoculum, with 23% of infectivity remaining in the phagostimulant granular viral formulation after 8 days, compared to <1% in a viral aqueous spray formulation. In addition, wettable powder formulation by microencapsulation of OBs protected the OBs against inactivation when exposed to UV-B radiation for 6 h in the laboratory, in contrast to unformulated OB suspension, which lost more than 50% of its original activity remaining (OAR) within the same exposure time [151].

To develop a potent SfMNPV and/or SfGV isolate into a commercial product, mass production of the OBs is essential. The technical difficulties and costs related to the production and formulation of these OBs is a major challenge limiting the commercialization of baculovirus isolates [32,35,64,138]. First, it is challenging to keep a healthy mass rearing of insects as they may be challenged by different pathogens, including (baculo) viral outbreaks resulting from covert virus infections. Therefore, rearing conditions are very important and must be optimal to maintain a healthy colony. In addition, the viral particles are produced in vivo, requiring large numbers of larvae to produce the OBs. The cannibalistic behavior of some species, including FAW, during mass rearing also constitutes a major challenge, reducing the number of larvae to produce OBs. Finally, the liquefaction of the integument makes the handling and recovery of the OBs challenging, constituting a major limitation in large-scale and commercial production [32,34,35]. For example, an SfMNPV commercial isolate was temporarily discontinued in Brazil due to the high cost of production arising from the aforementioned challenges after successful application on over 20,000 ha [32,40]. The problems related to mass production of the OBs can be addressed by freezing liquified larvae or collecting moribund larvae before liquefaction, which requires appropriate timing. However, this could potentially be more labor-intensive and time consuming. Another alternative is to collect SfMNPV isolates that do not cause liquefaction in the infected larvae, for example, natural isolates that lack genes encoding chitinase and cathepsin [77,157]. An SfMNPV isolate (SfMNPV-6) from Brazil that does not cause liquefaction of the integument has been characterized [77]. Sequencing the *chitinase A* (*v-chiA*) gene of SfMNPV-6 revealed a frameshift mutation that reduced the size of the putative enzyme [77]. When compared to a previously described virulent isolate (SfMNPV-19), SfMNPV-6 is equally as effective as SfMNPV-19 in terms of the lethal concentration against second instar larvae of FAW, although it takes a longer time (16.9 h difference of the mean survival time) to kill the larvae [77]. Therefore, isolate SfMNPV-6 has been selected for commercial formulation in Brazil (Table 3) [35]. In vitro production of baculoviruses in insect cell cultures offers another alternative for commercial mass production of OBs since it is more controllable, sterile, and will result in the yield of highly pure products. However, a major concern of this technology is the mutation of the isolates since several passages in insect cell cultures might lead to deletions in some parts of the genome, which might render the isolates less virulent [32,64,76] or even loss of oral infectivity [114]. Nonetheless, ongoing studies on the optimization of the production of baculovirus OBs in cell culture [158,159,160] showed that the propagation of SfMNPV in Sf9 and Sf21 cells in both static and suspension cultures results in a higher OB production in comparison to other cell types [159,160]. Further optimization of in vitro production in cell culture might lead to a successful commercial mass production. Another major challenge in using baculoviruses for biological control is the development of resistance in field populations. Different types of resistance of host populations against a baculovirus have been found in codling moth larvae, *Cydia pomonella*, against commercial formulations [161,162,163,164]. The different types of resistance of *C. pomonella* to CpGV were found to be isolate dependent and included different commercial formulations of various CpGV isolates. Whole genome sequencing-based analysis of CpGV isolates has shown that field-collected as well as commercially available CpGV isolates are mixtures of genotypes with different resistance-breaking properties [165,166], underlying the importance of the genotypic composition in baculovirus isolates. The only known case of resistance of FAW against an SfMNPV isolate is described by Fuxa et al. [167]. Resistance developed in FAW larvae after seven generations of constant selection pressure. The median lethal dose (LD_50_) increased 4.5-fold, from 4.1 to 18.7 OBs/insect in the laboratory colony exposed to the constant selection pressure when compared to the unchallenged colony (5.9 OBs/insect). However, the resistance was unstable and was reversed eight generations after the selection pressure was removed [168]. Nonetheless, resistance may evolve quickly when applying SfMNPV in the field against FAW. Further studies into the evolution of resistance and on management strategies to reduce the risk of resistance evolution are certainly warranted. Alternating the application of different SfMNPV isolates can improve the overall control efficacy and potentially delay resistance development.

The main social challenge of applying baculoviruses for biological control is the willingness of farmers to use viruses to control pests in the field [169]. The willingness varies amongst different geographical regions and amongst socio-economic groups of farmers [34,169,170,171]. Biological control with baculoviruses has been successfully adopted and used for several decades in North and South America [35,172], where there are more commercially based large estate farmers. In Europe, CpGV commercial isolates are widely used in orchards [172]. Similarly, in South Africa, Cryptophlebia leucotreta granulovirus (CrleGV) has been extensively used to control the false codling moth in citrus orchards [173]. In addition, OrNV, a nudivirus isolate, is also successfully deployed in many Asian countries to control the rhinoceros beetle that causes devastating damage to oil palm and coconut plantations [87]. Some farmers are, however, hesitant to “spray a virus” to control a pest in the field. Moreover, there could be real concerns about the possible downsides of the adoption of baculoviruses as biopesticides, such as their high cost in relation to chemical pesticides [170,171,174,175], and their variable performance in the field [103,138]. In addition to the points highlighted above, other possible reasons for the farmers’ hesitation are likely due to the limited understanding of the mode of action and safety of insect-pathogenic viruses, as well as the role viruses play in global pandemics [169,176]. More awareness is needed on the safety of baculovirus applications for farmers and the general public to increase the acceptance of baculovirus technology [32,170,176]. Local and international agricultural institutions, such as Food and Agriculture Organization (FAO), Centre for Agriculture and Bioscience International (CABI), International Institute of Tropical Agriculture (IITA), and International Centre of Insect Physiology and Ecology (ICIPE), play an important role in creating and increasing the awareness for the adoption and acceptance of the technology.

## 5. Conclusions

As FAW continues to spread into new territories, moving farther into the Eastern hemisphere and more recently to the Canary Islands of Spain, timely detection via adequate monitoring and surveillance is crucial to forestall the pest. Baculoviruses remain the most promising viral agent for biological control and finding local isolates of SfMNPV and SfGV is crucial to achieving a sustainable and effective control of local populations of FAW. Combinations of more than one SfMNPV/SfGV isolate could solve one of the main challenges of the use of viruses (reduced efficacy compared to chemical pesticides) and possibly help to delay the evolution of resistance. In addition, the combination of a mixture of different baculovirus isolates from different hosts could improve their prospect to control more than one pest species. Such mixtures could also help to limit cost and energy requirements by reducing the number of times required to spray the fields to control multiple pests, thereby giving similar benefits as chemical pesticides without the added side effects. However, consideration should be taken to ensure that such a mixture does not compromise the efficacy of the individual products. Future research should also focus on exploring the virome of *S. frugiperda*, which might provide valuable insight and information into the potential antagonistic and synergistic viruses present in the virome and the role they play in the host immune response. Widening the IPM scope through the combination of different control strategies, including viruses, will play a major role to manage the pest and keep the damage threshold below the economic injury level.

## Figures and Tables

**Table 1:** Virus families associated with FAW, host stage infected, symptoms, and key references.

Viruses Associated with FAW	Virus Name	Host Stage Infected	Main Symptoms	Key References
**DNA Viruses**				
Ascoviruses	Spodoptera frugiperda ascovirus (SfAV-1a)	Larvae	Stunting of infected larvae, production of virus filled vesicles, milky-white discoloration, fat body cells infection	[39,43]
Baculoviruses	Spodoptera frugiperda multiple nucleopolyhedrovirus (SfMNPV), Spodoptera frugiperda granulovirus (SfGV), Spodoptera littoralis nucleopolyhedrovirus (SpliNPV)	Larvae	Whitish-grey discoloration, swollen body, ruptured integument leading to liquefaction of the larvae	[34,35,50]
Densoviruses	Junonia coenia densovirus (JcDV)	Larvae	Anorexia, lethargy, hypoxia, and inhibition of molting	[42,51,52]
**RNA Viruses**				
Rhabdoviruses	Spodoptera frugiperda rhabdovirus	Sf9 and Sf21 cells	No described symptoms	[53]
Partiti-like viruses	Spodoptera exempta virus 1, 2, and 3 (SEIV1-3)	Larvae	Reduced the growth rate and fecundity of FAW larvae and increase susceptibility to baculovirus	[54]

**Table 2:** Host specificity of *Spodoptera* spp. baculoviruses found in the genus *Alphabaculovirus* [61,63,69,70,71].

	SfMNPV	SeMNPV	SpltNPV	SpliNPV
*S. frugiperda*	+++			+++
*S. exigua*	+	+++		+++
*S. litura*			+++	
*S. littoralis*	+			+++

+++ = permissive; + = semi permissive.

**Table 3:** Baculoviruses isolated from *S. frugiperda*.

Baculovirus	Isolate	Plaque Purified Isolate/Genotype	Country of Collection	Key Reference
SfMNPV	1	1BP2	USA	[104]
	2	2AP2	USA	[104]
	3	3AP2	USA	[104]
	4	4AP2	USA	[104]
	5	5AP1	USA	[104]
	6	6AP1	USA	[104]
	01 to 22		Brazil	[78,112]
	6 nd		Brazil	[77]
	SfHub	SfHub-A, SfHub-E	China	[108]
	M	M1, M11	Argentina	[113]
	C		Argentina	[113]
	SfNIC	SfNIC-A to SfNIC-I	Nicaragua	[76,114]
	SfCol	SfCol-A to SfCol-G	Colombia	[76,115]
	281, 637, 638, 651, 652, 653, 654, 1197, 2507, 3146		USA	[79]
	459, 635, 636		Colombia	[116]
	SfCH1, SfCH4, SfCH6, SfCH12, SfCH15, SfCH18, SfCH30, SfCH32		Mexico	[105,117]
SfGV	ARG		Argentina	[118]
	VG008		Colombia	[119]

**Table 4:** Countries that have registered baculoviruses able to infect FAW.

Product Name	Trademark	Baculovirus Isolate	Country of Registration	Key References
Baculomip-SF	Promip Manejo Integrado de Pragas Ltd.a	SfMNPV	Brazil	[125]
Baculonat SF	Bionat Soluções Biologicas Ltd.a	SfMNPV	Brazil	[125]
CartuchoVIT	Grupo Vitae Ltd.a—ME	SfMNPV-6	Brazil	[35,73]
Cartugen Cartugen CCAB	Agbitech Controles Biológicos Ltd.a	SfMNPV-3AP2	Brazil	[72,73]
Fawligen	AgBiTech Pty Ltd.	SfMNPV-3AP2	Bangladesh, Kenya, Sri Lanka, USA, Zambia, Australia	[72,74,75]
Laphy Protection	Biome Industry Commerce and Distribution—EIRELI	SfMNPV	Brazil	[125]
Littovir	Andermatt Biocontrol	SpliNPV	France, Portugal, Tunisia, Bulgaria, Spain, Italy, Morocco, Cameroon	[80,81,126,127]
Lungo	Agbitech Controles Biológicos Ltd.a	SfMNPV	Brazil	[125]
Spobiol	Colombian Agricultural Research Corporation	SfMNPV		[74]
Spodovir Plus	Andermatt Biocontrol	SfMNPV-6 and -19	Brazil, Paraguay	[125,128]
Surtivo Plus Surtivo Ultra	AgBiTech Pty Ltd.	Autographa californica multiple nucleopolyhedrovirus (AcMNPV), Chrysodeixis includens (ChinNPV), Helicoverpa armigera HearNPV and SfMNPV	Brazil	[73,74]
VirControl SF	Symbiosis Industry and Trade of Fertilizers and Microbiological Inputs Ltd.a	SfMNPV-6	Brazil	[35,73,74]
Vir Protection	Biome Industry Commerce and Distribution—EIRELI	SfMNPV	Brazil	[74,125]

## Data Availability

Publicly available datasets were analyzed in this study. Available online: https://www.ncbi.nlm.nih.gov/ (accessed on 6 June 2021).
